# Joint cartilage thickness measured by ultrasound in juvenile idiopathic arthritis

**DOI:** 10.1186/1546-0096-10-S1-A35

**Published:** 2012-07-13

**Authors:** D Pradsgaard, AH Spannow, CW Heuck, A Hørlyck, T Herlin

**Affiliations:** 1 Aarhus University Hospital, Skejby, Aarhus N, Region Midt, Denmark; 2 Aarhus N, Region Midt, Denmark

## Purpose

Joint cartilage degradation is one of the early outcomes in juvenile idiopathic arthritis (JIA). One of the primary goals of rheumatic treatment is to prevent joint destruction. We therefore need sensitive methods to evaluate cartilage thickness. Ultrasonography (US) has shown to be a reliable tool in the assessment of joint cartilage in rheumatic disease. We have previously been able to establish age- and sex-related normal reference intervals for joint cartilage thickness throughout childhood^(1)^. The purpose of this study was to investigate: - whether US is a sensible method in the evaluation of joint cartilage in JIA patients; - US feasibility as a monitoring and prognostic tool.

## Methods

In a cross-sectional study we included 49 patients diagnosed according to the 2001 revised ILAR classification of JIA. Subsets of JIA were 9 systemic onsets, 21 oligoarticular (P/E: 15/6), and 19 polyarticular (RF -/+: 14/5) JIA. The patients were all Danish Caucasians, mean age (range) 10.3 yrs (5-15), boys/girls: 17/32. A group of healthy children from a previous study [1] were used for comparison, 394 Danish Caucasians, age 10.9 (6-16), boys/girls: 217/177. Patients were examined with bilateral grey-scale US of knee joint cartilage. All measurements were made twice and the average was calculated. Knee cartilage was measured on the femur at the midline of the intercondylar notch, with knee maximally flexed. Scans were based on EULAR standard scans.

## Results

The age, height, and weight, did not differ between JIA subtypes and healthy children (ANOVA: p=0.4, p=0.2, p= 0.3). Disease duration did not differ between JIA subtypes (p= 0.3). We found a significant difference in the average knee cartilage thickness between oligo- (3.0 mm ±0.7) and poly- (2.5 mm ±0.5) JIA, also when controlling for age and sex (linear regression: p<0.01). There was a significant difference between healthy (3.5 mm ±0.5) and all three JIA subgroups (Systemic onset: 2.8 mm ±0.6, Oligo- 3.0 mm ±0.7, Polyarticular 2.5 mm ±0.5) when controlling for age and sex, (lin. regress: p< 0.001 for all). Knee cartilage were significantly thicker in JIA boys (3.1 mm ±0.6) than JIA girls (2.6 mm ±0.6) (Mann-Whitney, p<0.005)) and in healthy boys (3.7 mm ±0.5) compared to girls (3.3 mm ±0.5) (p< 0.001). There was a significant difference between right (2.7 mm ±0.6) and left (2.9 mm ±0.7) side within all JIA patients(Paired t-test, p=0.01), but no difference within the healthy group. Cartilage thickness decreased with increasing age in all groups, but not with increasing duration of disease.

## Conclusion

We found differences in knee cartilage thickness between oligo- and polyarticular JIA and between healthy children and all three subtypes of JIA. In the present study we also found a difference between sexes as previously shown in the healthy control group^(1)^, but in contrast we did find a difference between right and left knee. Our results shows that US is able to assess even small changes in joint cartilage thickness, which makes it valuable in the evaluation of joint diseases in childhood.

## Disclosure

D. Pradsgaard: None; A. H. Spannow: None; C. W. Heuck: None; A. Hørlyck: None; T. Herlin: None.
